# The Curious Case of Confounding Headaches

**DOI:** 10.1155/crrh/2146010

**Published:** 2025-09-27

**Authors:** Ram Chandra Khatri Chhetri, Hemanta Paudel, Viswaja Kaja, Jahanzeb Saeed, Jane Nwaonu, Adegbenga Bankole

**Affiliations:** ^1^Carilion Clinic, Roanoke, Virginia, USA; ^2^Carilion Roanoke Memorial Hospital, Roanoke, Virginia, USA

## Abstract

Giant cell arteritis is the most common primary systemic vasculitis among individuals over 50 years of age. It primarily affects large- and medium-size arteries and is not mediated by antibodies. One of the most recognizable and important symptoms of the disease is headache. The presence of headaches, along with other common cranial manifestations such as vision loss, jaw claudication, and scalp tenderness in the temporal arteries, can assist in diagnosing the condition. We present a complex case involving a 76-year-old male with prolonged headaches, a pituitary macroadenoma, and vestibular schwannoma. Initially, his headaches were attributed to his existing intracranial lesions; however, his symptoms continued to evolve. He continued to have headaches of varying intensity over 2 years, and subsequently developed diffuse scalp tenderness, visual disturbances, and tongue claudication. Input from various medical specialties expanded the differential diagnosis and raised the possibility of giant cell arteritis (GCA). Although the temporal artery biopsy did not reveal the classic giant cells typically associated with the condition, it supported the clinical diagnosis of GCA. Appropriate treatment with high-dose corticosteroids and anti-Interleukin 6 therapy resulted in the rapid resolution of his symptoms. This case emphasizes the importance of recognizing different types of headaches, maintaining a broad differential diagnosis, and thoroughly evaluating all clinical symptoms for timely diagnosis and treatment. It also highlights the significance of a multidisciplinary approach to ensure prompt diagnosis and to prevent irreversible complications, such as permanent vision loss.

## 1. Introduction

Giant cell arteritis (GCA) is a granulomatous vasculitis that predominantly affects large-size and medium-size arteries, typically occurring in women over the age of 50 [1]. It is more prevalent in Western populations, estimated at 15–25 per 100,000 per year, with prevalence increasing with age [2]. Individuals of Caucasian origin, especially those from Scandinavian and Northern Europe, are commonly affected [3]. Multiple studies have found that new-onset headache is the most prevalent clinical feature of GCA, occurring in approximately 67%–75% of patients [4, 5]. Although temporal headaches are the classical presentation, the symptomatology in individual patients may be heterogeneous, and this can lead to diagnostic challenges [6]. Even when headaches exhibit typical characteristics of GCA, the presence of other diseases that can present with headaches may lead to significant delays in diagnosis [7]. In such situations, other manifestations such as jaw claudication, scalp tenderness, fever, malaise, and weight loss can help differentiate GCA from other noninflammatory pathologies [8].

In this case, we explore the diagnostic journey of a 76-year-old male with a complex neurological history, including vestibular schwannoma and pituitary macroadenoma, who presented with persistent and evolving headaches. This case highlights the importance of considering GCA in elderly patients with new-onset and evolving headaches and the necessity of a multidisciplinary approach to diagnosis and management to prevent severe complications.

## 2. Case Presentation

A 76-year-old male presented initially to his primary care team with swelling, pain, and a burning sensation in his tongue. He also reported a right-sided dull and achy headache without migraine aura, nausea, and vomiting. His headaches were described as diffuse with shooting pains and scalp hypersensitivity. He did note some balance difficulties including feeling unsteady while climbing stairs but not while walking straight. He also noted decreased hearing and the sensation of bilateral ear fullness.

**Figure 1:**
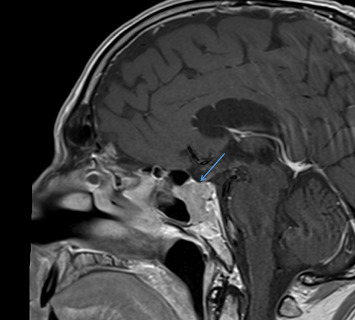
Sagittal MRI brain with and without contrast. Blue arrowhead indicates anterior sella mass involving the anterior sella and clivus without optic chiasm compression with extension to the level of the cavernous sinuses (picture taken with consent).

He was evaluated by the Ear, Nose, and Throat (ENT) team, confirming right-sided progressive hearing loss on an audiogram. He was noted to have a normal complete blood count (CBC), complete metabolic panel (CMP), vitamin B12, folate, zinc, and thyroid-stimulating hormone (TSH) levels. Magnetic resonance imaging (MRI) showed a mass involving the anterior sella and clivus without optic chiasm compression (Figure 1). The differential diagnosis of this mass included pituitary adenoma, chordoma, or metastatic disease, and this directed further workup, and several hormonal tests were performed (Table 1). An ophthalmology examination confirmed normal-looking eyelids, bulba, cornea, and sclera, with a deep and quiet anterior chamber and normal iris. A slit lamp examination was normal, as was the retina.

**Figure 2:**
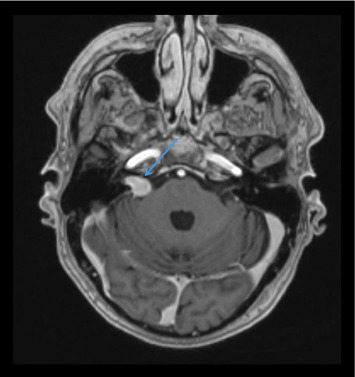
Axial reconstruction postcontrast MRI. White arrowhead shows right vestibular schwannoma with intracanalicular and cisternal components. No significant mass effects on the adjacent brainstem or middle cerebellar peduncle.

Chest, abdomen, and pelvis computed tomography (CT) did not show a malignancy. A repeat MRI confirmed a 1.6-cm right vestibular schwannoma and the anterior sella mass seen in previous MRI (Figure 2).

He was evaluated by neurology and underwent gamma knife radiosurgery. Despite this intervention, his headache, balance, and hearing issues persisted. He noted some improvement in his facial weakness or numbness. He did receive amitriptyline and two occipital nerve blocks with no significant improvement in his headaches.

He continued to have headaches, and about 2 years after initial presentation, he developed vision changes described as “shards of glass” in the right visual field. He also noted a change in the headaches with increased intensity, more diffuse headaches, and profound fatigue, prompting an emergency department (ED) visit. A repeat CT head did not reveal any new changes, and other than his inflammatory markers, his laboratory investigations were unremarkable (Table 2).

Bilateral temporal artery biopsies were performed and showed prominent lymphohistiocytic inflammation within intima and media, scattered foci of neutrophilic and eosinophilic inflammation, arterial wall thickening, elastic fiber fragmentation, and focal fibrinoid wall necrosis, consistent with temporal arteritis. Although definitive giant cells and granulomas were not identified, the findings confirmed the diagnosis of temporal arteritis.

He was treated with 1000 mg of intravenous methylprednisolone daily for 3 days and discharged on oral prednisone 60 mg daily. At his follow-up visit in the rheumatology clinic, he noted complete resolution of headaches, scalp tenderness, and jaw claudication, although tongue claudication persisted initially. Tocilizumab, an anti-Interleukin 6 receptor antibody, was initiated as a steroid-sparing agent. He started to taper the prednisone once he got his intravenous IL-6 therapy. Ultimately, the prednisone was tapered successfully, without recurrence of his symptoms, and he remained stable without major complications from his complex neurological history in subsequent follow-up visits at the rheumatology clinic.

## 3. Discussion

GCA is systemic vasculitis that may pose significant diagnostic and therapeutic challenges, particularly in elderly patients with complex neurological histories. Our case of a 76-year-old male illustrates the intricacies of diagnosing GCA amidst differential diagnoses with overlapping symptoms, as in this case with vestibular schwannoma and pituitary macroadenoma.

Our patient had some symptoms not attributed to GCA, including loss of hearing, difficulties in balance, visual distortion of the near field related to his vestibular schwannoma, and pituitary macroadenoma, complicating the diagnostic process. His headache, although different from what is generally seen in both vestibular schwannoma and pituitary macroadenoma, was still thought to be related to one or both of these conditions. In vestibular schwannoma, headache typically arises from cerebellopontine angle compression and is usually described as dull, persistent, and localized to the retroauricular or occipital area [9]. Similarly, pituitary microadenomas can cause headaches due to stretching of the dura mater or cavernous sinus invasion, which often presents as bifrontal headache accompanied by visual field deficits or hormonal abnormalities [10]. These headaches are less likely to be associated with systemic symptoms such as scalp tenderness, jaw claudication, and tongue necrosis that can be seen in GCA. His persistent headaches were initially attributed to his pre-existing conditions and delayed the diagnostic journey toward GCA.

GCA is present in 75.7% of patients with new-onset headache, and is the hallmark of GCA [3]. GCA should be considered in people within the right demographics for GCA, with new-onset headaches, or changes in the nature and quality of headache symptoms in those with illnesses that may also cause headaches. Our patient initially experienced unilateral and stabbing headaches that became bilateral, eventually diffused with scalp sensitivity. This progression was atypical of his prior headaches, attributed to his vestibular schwannoma and pituitary macroadenoma, and was different from his prior migraines. The absence of his typical migraine headache features eventually raised suspicion of a systemic cause. The presence of additional symptoms such as elevated inflammatory markers, tongue claudication, and vision changes further pointed toward GCA [6]. Initial imaging revealed the vestibular schwannoma and pituitary macroadenoma, which could have contributed to the headaches. However, the persistence and evolution of symptoms necessitated further investigation. A repeat MRI was performed, and this did not indicate progression of his intracerebral lesions. Imaging tests such as scalp ultrasound and MRI of temporal vessels can be diagnostic [6]. Within the United States of America, a temporal artery biopsy remains the gold standard for diagnosing GCA [7]. In this case, bilateral biopsies confirmed the diagnosis of GCA. Our patient highlights the importance of the medical community being aware that the presences of giant cells are not required to make the diagnosis [8].

Once the diagnosis is made, prompt initiation of corticosteroids is crucial to prevent severe complications, such as irreversible vision loss [4]. Our patient received high-dose methylprednisolone, followed by prednisone, leading to the resolution of headaches and scalp tenderness. The management of GCA also involves the use of immunosuppressive medications, including Interleukin 6, monitoring for side effects and a careful plan for tapering and discontinuation of prednisone [5]. Close collaboration between neurology, neurosurgery, rheumatology, and ophthalmology was essential in this case to address all aspects of the patient's health and ensure comprehensive care.

## 4. Conclusion

This case emphasizes the diagnostic difficulties and the critical importance of identifying GCA in patients who have persistent yet changing headaches, especially when they also have pre-existing neurological conditions that cause headaches. Clinicians should maintain a high level of suspicion for GCA, even in patients with other neurological diagnosis, if not all symptoms are adequately explained. The complexity of this situation highlights the necessity of a multidisciplinary approach in managing patients with symptoms that could be attributed to multiple overlapping conditions. Such an approach is crucial in complicated cases to reduce the risk of increased morbidity and mortality associated with diagnostic delays.

## Figures and Tables

**Table 1:** Laboratory tests.

Labs	Value	Reference range
Testosterone, total male (adult) immunoassay	388	250–827 ng/dL
Sex hormone–binding globulin	41	22–77 nmol/L
Free testosterone	42.3	6.0–73.0 pg/mL
Bioavailable testosterone	85.2	15.0–150.0 ng/dL
Thyroid-stimulating hormone	1.279	0.55–4.78 uIU/mL
Triiodothyronine	151	60 - 170 NG/dL
Insulin like growth Factor-1 liquid chromatography mass spectrometry	116	34–245 ng/mL
Adrenocorticotropic hormone	41	6–50 pg/mL
Growth hormone	< 0.1	≤ 7.1 ng/mL
Follicle-stimulating hormone	8.6	1.6–8.0 mIU/mL
Cortisol	13.88	3.44–16.76 UG/dL
Prolactin	5.1	2.0–18.0 ng/mL
Luteinizing hormone	3.42	3.1–34.6 mIU/mL

**Table 2:** Laboratory investigations.

Component	Results	Reference range
White blood cell	7.6	4.0–10.5 K/uL
Hemoglobin	12.3	12.0–16.0 g/dL
Hematocrit	37.2	37%–49%
Platelets	393	130–400 K/uL
Urea nitrogen	19	7–23 mg/dL
Creatinine	0.81	0.70–1.30 mg/dL
Albumin	4.1	3.2–5.0 g/dL
Alkaline phosphatase	86	46–116 U/L
AST	29	15–37 U/L
ALT	19	10–49 U/L
Sed rate	**65 (H)**	0–20 mm/HR
C-reactive protein	**2.70 (H)**	< 1.0 mg/dL

*Note:* The Sed Rate and C-reactive protein are elevated indicating systemic inflammation.

## Data Availability

All relevant data are available on request due to privacy/ethical restrictions.
